# Gender associates with both susceptibility to infection and pathogenesis of SARS-CoV-2 in Syrian hamster

**DOI:** 10.1038/s41392-021-00552-0

**Published:** 2021-03-31

**Authors:** Lunzhi Yuan, Huachen Zhu, Ming Zhou, Jian Ma, Rirong Chen, Yao Chen, Liqiang Chen, Kun Wu, Minping Cai, Junping Hong, Lifeng Li, Che Liu, Huan Yu, Yali Zhang, Jia Wang, Tianying Zhang, Shengxiang Ge, Jun Zhang, Quan Yuan, Yixin Chen, Qiyi Tang, Honglin Chen, Tong Cheng, Yi Guan, Ningshao Xia

**Affiliations:** 1 State Key Laboratory of Molecular Vaccinology and Molecular Diagnostics, National Institute of Diagnostics and Vaccine Development in Infectious Diseases, School of Life Sciences, School of Public Health, Xiamen University, Xiamen, P. R. China; 2 State Key Laboratory of Emerging Infectious Diseases, The University of Hong Kong, Hong Kong, P. R. China; 3 Joint Institute of Virology (Shantou University and The University of Hong Kong), Guangdong-Hongkong Joint Laboratory of Emerging Infectious Diseases, Shantou University, Shantou, P. R. China; 4 Department of Microbiology, Howard University College of Medicine, Washington, DC USA; 5 Research Unit of Frontier Technology of Structural Vaccinology, Chinese Academy of Medical Sciences, Xiamen, Fujian China

**Keywords:** Infectious diseases, Respiratory tract diseases

## Abstract

Epidemiological studies of the COVID-19 patients have suggested the male bias in outcomes of lung illness. To experimentally demonstrate the epidemiological results, we performed animal studies to infect male and female Syrian hamsters with SARS-CoV-2. Remarkably, high viral titer in nasal washings was detectable in male hamsters who presented symptoms of weight loss, weakness, piloerection, hunched back and abdominal respiration, as well as severe pneumonia, pulmonary edema, consolidation, and fibrosis. In contrast with the males, the female hamsters showed much lower shedding viral titers, moderate symptoms, and relatively mild lung pathogenesis. The obvious differences in the susceptibility to SARS-CoV-2 and severity of lung pathogenesis between male and female hamsters provided experimental evidence that SARS-CoV-2 infection and the severity of COVID-19 are associated with gender.

## Introduction

Current pandemic of severe acute respiratory syndrome coronavirus 2 (SARS-CoV-2) has been causing a rapid increase of cases of coronavirus disease 2019 (COVID-19), which has been exerting a devastating harm to public health, economy, and social communication worldwide. Because lung is the main target organ of SARS-CoV-2, typical clinical symptoms include fever, cough, and weakness that were observed in ~80% of COVID-19 patients, and 20% of them develop respiratory difficulties and organ failures.^1,2^ The pathological changes of lung include pneumonia, alveolar septum consolidation, and pulmonary edema among the severe cases. The outcomes of SARS-CoV-2 infection, and the progression and consequences of COVID-19 are complicated. The mechanisms of how SARS-CoV-2 causes different degrees of symptoms are unclear. Clinically, risk factors such as gender, age, and underlying diseases might be related to the severity of COVID-19. Several recent cohort studies have suggested that SARS-CoV-2 infection causes more severe lung diseases and higher mortality in male than in female.^3–6^ It was also found that males are more susceptible to SARS-CoV infection than females by epidemiological studies^7,8^ and animal studies in mice.^9^ Therefore, investigating the gender bias in SARS-CoV-2 infection will improve our fundamental understanding of the pathogenesis of COVID-19 and help the development of prevention strategies of SARS-CoV-2 infection. However, a detailed pathological study regarding gender bias of COVID-19 in an animal model is still lacking. Recently, an outbred rodent strain, Syrian hamster, has been demonstrated to be a susceptible animal mode for SARS-CoV-2 infection.^10–12^ Hamsters showed rapid loss of body weight, viral shedding in nasal washings and lung tissue injury throughout a 7-day SARS-CoV-2 infection course.^10–12^ In this study, we applied the Syrian hamster as a model to not only study the pathogenesis of SAR-CoV-2 infection but also investigate whether gender could play a role in SARS-CoV-2 infection.

## Results

### Symptoms and viral shedding in male and female hamsters post SARS-CoV-2 infection

**Fig. 1:**
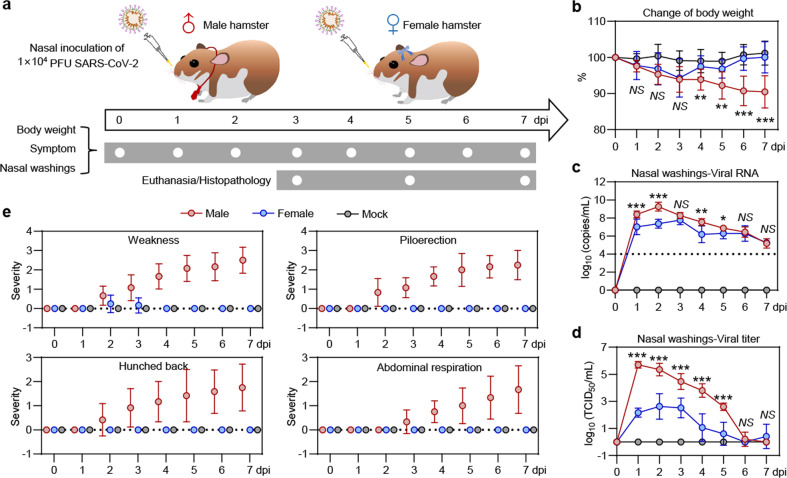
Symptoms and viral shedding of male and female hamsters that were intranasally inoculated with SARS-CoV-2. **a** Schematic diagram of SARS-CoV-2 infection and animal operations. Male and female hamsters were intranasally inoculated with 10^4^ PFU of SARS-CoV-2. Body weight and symptoms were daily examined. Nasal washings were daily collected. **b** Change of body weight (*n* = 12/group). **c** PCR detection for viral RNA levels in nasal washings (*n* = 6/group). **d** Titration for virus levels in nasal washings (*n* = 6/group). **e** Daily observation of typical symptoms including weakness, piloerection, hunched back, and abdominal respiration (*n* = 12/group). Each symptom was scored based on the severity of none (0), moderate (1), mild (2), severe (3), and very severe (4), respectively. Detailed data of individual animals were shown in Supplementary Figs. 1 and 3

To investigate whether the gender could be a factor affecting the infection and pathogenesis of SARS-CoV-2, male and female adult Syrian hamsters were intranasally inoculated with 1 × 10^4^ plaque-forming unit (PFU) of SARS-CoV-2 (Fig. 1a). First, physical and health examinations were undertaken for a week to record their body weight and symptoms. The infected male hamsters exhibited progressive mean body weight loss of up to 9.6% from 1 to 7 days post infection (dpi) (Fig. 1b and Supplementary Fig. 1a–c). Whereas, the infected female hamsters and the mock-infected ones showed no significant body weight loss (Fig. 1b and Supplementary Fig. 1a–c). Then, the viral shedding in nasal washings was also measured. In comparison with the female hamsters, the males showed higher viral RNA load (Fig. 1c and Supplementary Fig. 1d–f) and virus load (Fig. 1d and Supplementary Fig. 1g–i) in nasal washings from 1 to 5 dpi. These results suggested that male hamsters are more sensitive to SARS-CoV-2 infection and replication. The viral RNA load and viral titer reach a peak on 1 or 2 dpi. Recent studies revealed that adaptive immune responses to SARS-CoV-2 infection and production of SARS-CoV-2-specific neutralizing antibody might indicate the disease severity of COVID-19 patients.^13,14^ The serological experiments showed a robust increase of SARS-CoV-2 receptor-binding domain (RBD) specific IgG in serum in both male and female hamsters. However, the females showed a higher SARS-CoV-2 RBD-specific IgG than the males (Supplementary Fig. 2). More importantly, all the male hamsters developed certain degrees of symptoms include weakness, piloerection (or ruffled furs), hunched back posture, and abdominal respiration (rapid breathing) since 2 dpi (Fig. 1e, Supplementary Fig. 3, and Videos V1–V3). Whereas, these symptoms were rarely seen in female hamsters throughout the infection course. In summary, the infected male hamsters presented features that were similar to that in severe COVID-19 patients and the female hamsters acted like the “asymptomatic” carriers.

### Differential pathological abnormalities in lung caused by SARES-CoV-2 infection in between male and female hamsters

**Fig. 2:**
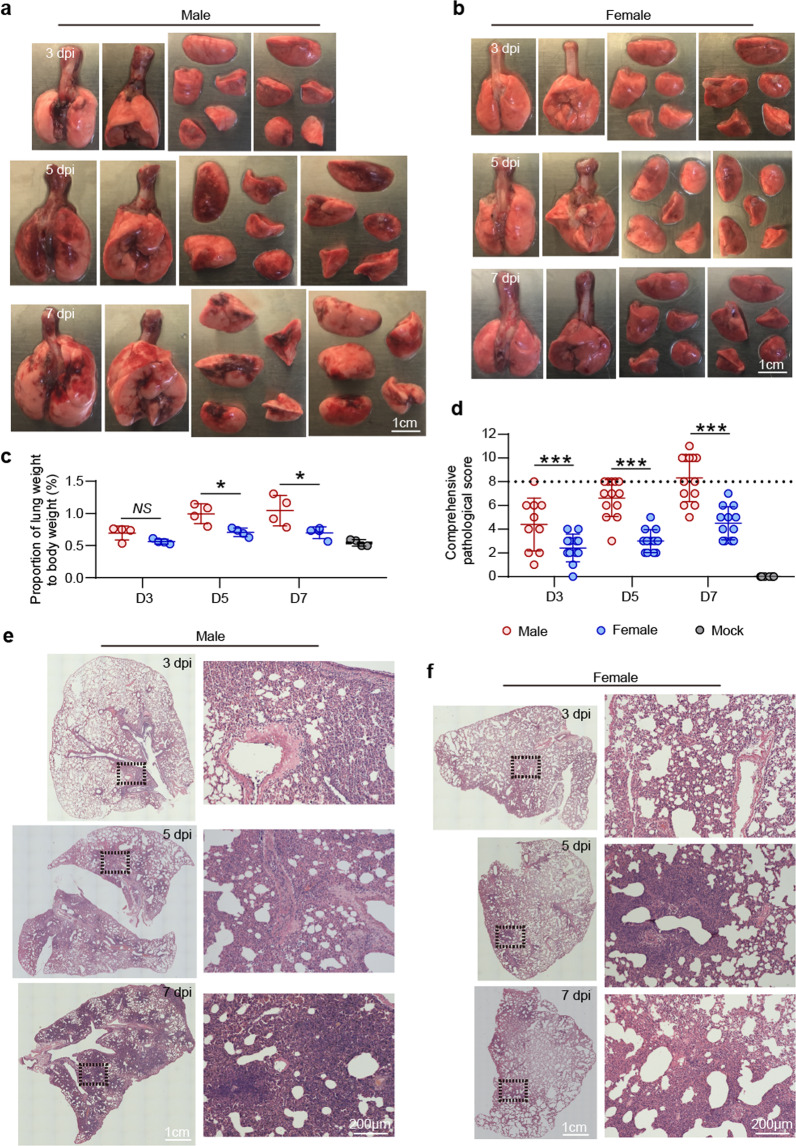
Pathological and histological analysis of the lungs from the SAES-CoV-2-infected hamsters. Gross observation of lung tissues collected from **a** male and **b** female hamsters that were nasally inoculated with 10^4^ PFU of SARS-CoV-2 at 3, 5, and 7 dpi, respectively (bar = 1 cm). **d** Ratio of lung weight to body weight of indicated male and female hamsters at 3, 5, and 7 dpi, respectively. Hamster without SARS-CoV-2 infection set as mock control (*n* = 4/group). **d** Comprehensive pathological score of lung sections derived from H&E staining for representative lung lobes collected from **e** male and **f** female hamster that are infected with SARS-CoV-2 at 3, 5, and 7 dpi. Views of the whole lung lobes were shown in left panels (bar = 1 cm) and areas in black box were enlarged in right panels (bar = 200 μm). Scores were determined based on the severity and percentage of injured areas for each lung lobe collected from indicated animal. Detailed images of more lung lobes were shown in Supplementary Figs. 5 and 6

To evaluate the pathogenesis of SARS-CoV-2 in lung, male and female hamsters were euthanized at 3, 5, and 7 dpi, and histological analyses were performed. First, we anatomized the animal and isolated the lungs from infected male or female hamsters as shown in Fig. 2a, b. Severe lung lesions include consolidation, multifocal, and diffuse hyperemia were seen in male hamsters at 5 and 7 dpi (Fig. 2a), but not in the female hamsters (Fig. 2b) or the mock animals (Supplementary Fig. 4). In addition, the ratio of lung weight to body weight or the ratio of the size of lung to the body size in male hamsters was significant increased at 5 and 7 dpi (Fig. 2c), which is consistent to the report from a study in rhesus macaques that were infected with SARS-CoV-2.^15^ These observations indicate a pulmonary edema caused by SARS-CoV-2 in male hamsters. To evaluate the pathogenesis caused by SARS-CoV-2 in lung, we performed the hematoxylin and eosin (H&E) staining for the lung sections collected from the infected male and female hamsters. Generally, the male hamsters showed more severe pathological lesions of lung than the females as summarized in Fig. 2d, Supplementary Figs. 5, 6, and Supplementary Table 1. The H&E experiments showed that the male hamster developed severe interstitial pneumonia and alveolitis diffused around the hilar areas at 3 dpi (Fig. 2e and Supplementary Fig. 5). An increasing lung lobe consolidation and alveolar destruction, diffuse inflammation, protein-rich fluid exudate, hyaline membrane formation, and severe pulmonary hemorrhage were observed throughout the whole lung lobes at 5 and 7 dpi (Fig. 2e and Supplementary Fig. 5). These lung pathological features were consistent with the autopsy results of the deceased severe COVID-19 patients.^16–18^ In the infected female hamsters, lung lesions exhibited multifocal, mild to moderate, interstitial pneumonia that scattered around terminal bronchioles at 3 and 5 dpi (Fig. 2f and Supplementary Fig. 6). Less than 20% of lung lobe consolidation with focal inflammation was observed at 7 dpi (Fig. 2f and Supplementary Fig. 6). Meanwhile, immunohistochemistry assay was performed to examine SARS-CoV-2 nucleocapsid protein (NP) and the results showed more abundant viral NP expression in male hamsters at 3 and 5 dpi (Supplementary Figs. 7–9). Interestingly, a rapid decreasing ratio of SARS-CoV-2 NP positive cell in lung tissues was detected in both male and female infected hamsters from 5 to 7 dpi (Supplementary Figs. 7–9). Overall, the male hamsters presented more severe pathological abnormalities than the females, suggesting that gender is a factor to affect the outcome of the SARS-CoV-2 infection in lung.

### A low infecting viral dose is enough to cause pathogenicity of SARS-CoV-2 in male hamsters

**Fig. 3:**
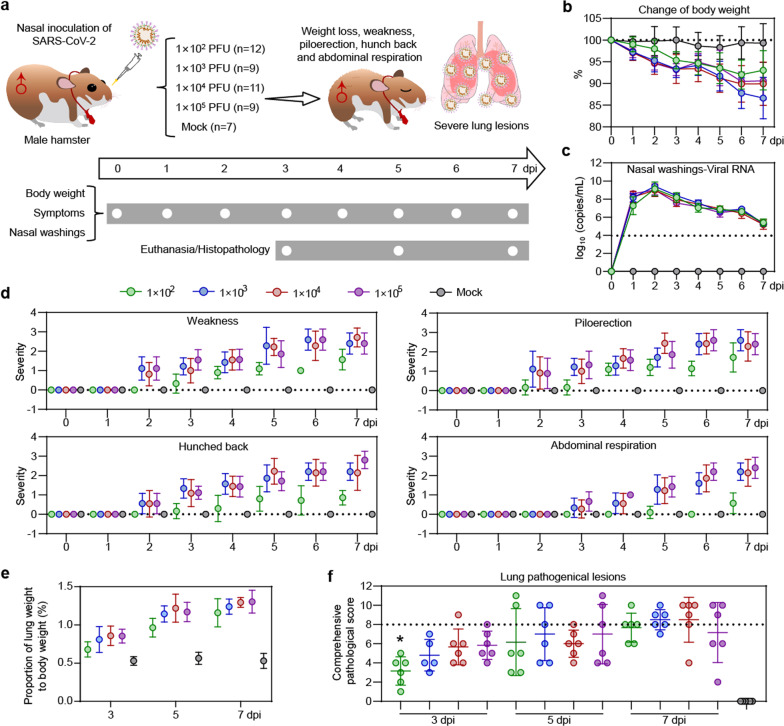
Symptoms and viral shedding in male hamster intranasally inoculated with varied doses of SARS-CoV-2. **a** Schematic diagram of SARS-CoV-2 infection and animal operations. Male hamsters were nasally inoculated with 10^2^–10^5^ PFU of SARS-CoV-2, respectively. Body weight and symptoms were daily observed. Nasal washings were daily collected. Animals were euthanized at 3, 5, and 7 dpi for lung histological analysis, respectively. **b** Change of body weight. **c** PCR detection for viral RNA levels in nasal washings. **d** Daily observation for typical symptoms includes weakness, piloerection, hunched back, and abdominal respiration. Each symptom was scored based on the severity of none (0), moderate (1), mild (2), severe (3), and very severe (4), respectively. Detailed data of individual animals in (**b**–**d**) were shown in Supplementary Figs. 10–12. **e** Ratio of lung weight to body weight of indicated male hamsters at 3, 5, and 7 dpi, respectively. Hamsters without SARS-CoV-2 infection were set as mock controls (*n* = 2/group). **f** Comprehensive pathological score of lung sections for lung sections collected from male hamster nasally inoculated with 10^2^–10^5^ PFU of SARS-CoV-2 at 3, 5, and 7 dpi, respectively. For each group, different lung lobes collected from two individual animals were collected at 3, 5, and 7 dpi, respectively. Detailed images of lung lobes were shown in Supplementary Fig. 16

**Fig. 4:**
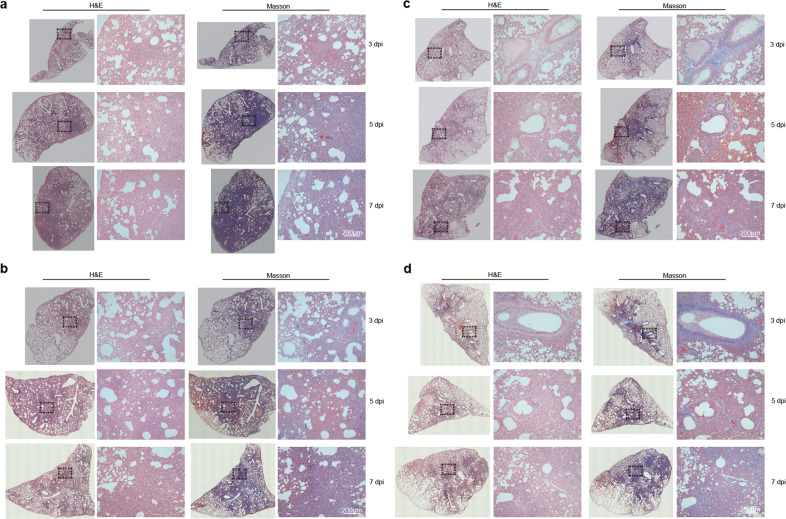
Pathological analysis of lung pathological lesions of hamsters nasally inoculated with different doses of SARS-CoV-2. H&E staining and Masson staining for representative lung sections collected from male hamsters nasally inoculated without **a** 1 × 10^2^, **b** 1 × 10^3^, **c** 1 × 10^4^, and **d** 1 × 10^5^ PFU of SARS-CoV-2 infection at 3, 5, and 7 dpi, respectively. Views of the whole lung lobes were shown in left panels and areas in black box were enlarged in right panels (bar = 200 μm)

**Fig. 5:**
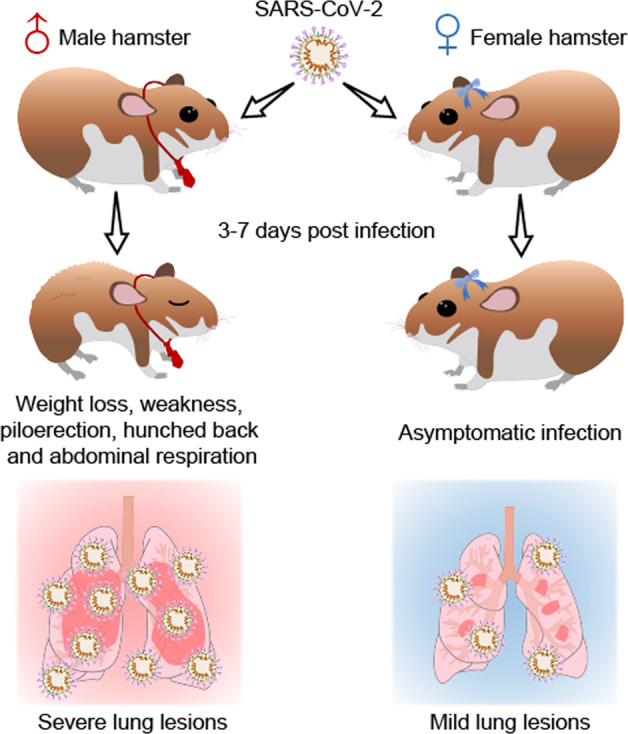
Schematic summary of the male gender bias of SARS-CoV-2 infection in hamster

We wondered how susceptible the male hamster is to SARS-CoV-2 infection. To that end, male hamsters were challenged with a gradient amount of SARS-CoV-2 from 1 × 10^2^ to 1 × 10^5^ PFU (Fig. 3a). Health and physical examinations were undertaken for 7 days to record the body weight and symptoms. We also measured viral shedding in nasal washing samples and performed histological analysis for the lung tissues of the infected animals that were euthanized at 3, 5, and 7 dpi. First, the male hamsters exhibited a body weight loss. Our results showed that the body weight loss reached 7.1%, 13.4%, 10.1%, and 9.4% in the groups of hamsters infected with 1 × 10^2^, 1 × 10^3^, 1 × 10^4^, and 1 × 10^5^ PFU at 7 dpi, respectively (Fig. 3b and Supplementary Fig. 10). No significant differences in the viral RNA load from the nasal washing samples from 1 to 7 dpi were detected among the different infection dose groups (Fig. 3c and Supplementary Fig. 11). In addition, the hamsters infected with 1 × 10^2^ PFU of SARS-CoV-2 developed mild symptoms, all other hamsters infected with 1 × 10^3^, 1 × 10^4^, or 1 × 10^5^ PFU of SARS-CoV-2 developed a similar degree of typical symptoms such as weakness, piloerection (or ruffled furs), hunched back posture and abdominal respiration (rapid breathing) (Fig. 3d and Supplementary Fig. 12). We did not detect any differences in levels of specific IgG against SARS-CoV-2 RBD among the animals infected with different amount of viruses (Supplementary Fig. 13). Similarly, no significant differences were seen for the presence of SARS-CoV-2 protein, NP in lung lobes of the hamsters that were infected with different amount of viruses (Supplementary Figs. 14 and 15). The ratios of lung weight to body weight for the male hamsters inoculated with 1 × 10^2^ to 1 × 10^5^ PFU of SAES-CoV-2 were significant increased at 5 and 7 dpi (Fig. 3e). Moreover, all of the male hamsters developed a similar degree of lung pathogenic changes at 3, 5, and 7 dpi, respectively (Fig. 3f, Supplementary Fig. 16, and Supplementary Table 2). Notably, 10^2^ PFU of SARS-CoV-2 is adequate to induce severe pneumonia, pulmonary edema, consolidation, and fibrosis (Fig. 4a). The results of H&E staining and Masson staining suggested that lung pathogenic lesions such as pneumonia and pulmonary consolidation are accompanied by abnormal collagen hyperplasia and accumulation from 3 to 7 dpi (Fig. 4, Supplementary Figs. 16 and 17). Consequently, alveolar destruction, diffuse inflammation, and fibrosis largely damaged the structure and respiratory function of the lung organ and induced the symptoms. Meanwhile, as important controls, female hamsters were challenged with a gradient amount of SARS-CoV-2 from 1 × 10^2^ to 1 × 10^5^ PFU. In contrast of the males, the female hamsters, especially for the ones with lower viral load inoculation showed no significant body weight loss and mild lung lesions (Supplementary Figs. 18, 19, and Supplementary Table 3). Taken together, our results demonstrated the male hamster is highly susceptible to SARS-CoV-2 infection to cause a disease like COVID-19 (summarized in Fig. 5).

## Discussion

Both epidemiological and clinical evidence revealed that males are more susceptible to SARS-CoV-2 infection causing COVID-19 than female.^19,20^ Although it was also studied in mice,^9^ a detailed pathological study regarding COVID-19 in an animal model regarding the sex and gender is still lacking. Our overarching outcomes of this study are that the male hamsters presented a clearly more susceptibility to SARS-CoV-2 infection and exhibited more severe symptoms of COVID-19 than females (Figs. 1 and 3). Interestingly, although the SARS-CoV-2 infection in both male and female hamsters caused viral replication, the replicated viral levels in females are much lower than in males (Fig. 1c, d). Similarly, the symptoms in SARS-CoV-2-infected male hamsters are as severe as COVID-19, but no significant symptoms were seen in female hamsters (Fig. 1b). Remarkably, the observations of the symptoms are consistent with the results of the pathogenic studies as shown in Figs. 2 and 4. Finally, we found that the female hamster showed a higher serum RBD-specific IgG levels than the males, indicating a stronger humoral immune response in the female plays a role in alleviating the progress of the diseases.

The mechanisms of how gender serves as a factor to affect the severity of COVID-19 might be more complicated than we currently understood. The male COVID-19 patients usually developed excessive innate immune responses with high levels of proinflammatory innate immune chemokines and cytokines such as IL-8, IL-18, and CCL5,^21^ which suggested a high risk of cytokine storm to cause tissue injury and organ failure. In contrary, the female COVID-19 patients showed appropriate innate immune responses and robust T-cell responses, especially for activation of CD8-positive T cells after SARS-CoV-2 infection.^21^ In addition, host immune responses are affected by hormone levels and other signaling,^22^ which might contribute to the severity of COIVD-19. These differences between the females and males might explain why the SARS-COV-2 infection causes more health problems in males than in females. Our animal studies provided an animal model for more detailed studies on how SARS-CoV-2 is more susceptible to males and causes more dire symptoms to males than to females. For instance, important factors such as age, expression level of ACE2, and specific host genes that might impact on SARS-CoV-2 infection, pathogenesis, and disease severity need to be investigated in future studies.

An appropriate animal model is important in studies of SARS-CoV-2 not only for developing antivirals, vaccines, and diagnoses but also for investigating how the virus infection causes COVID-19. Although many different kinds of animals have been reported to support SARS-CoV-2 infection, such as rhesus macaques,^15,23,24^ hACE2-transgenic mice^25^, and ferrets,^26,27^ they did not suffer the disease of COVID-19 in terms of the critical pathogenic progression and severe lung injury. For instance, most of such animal models developed mild interstitial pneumonia after SARS-CoV-2 infection. Severe and diffused lung lesions and relevant symptoms such as abdominal respiration was rarely observed. Thus, they may not be optimal for evaluation of risk factors that might affect the outcomes of SARS-CoV-2 infection and severity of lung pathogenesis. Hamster has been demonstrated sensitive to SARS-CoV infection^28^ and used to test SARS-CoV inactivated vaccine and therapeutic antibody.^29,30^ Recently, hamster was used for evaluation of SARS-CoV-2 countermeasures such as antibody and convalescent plasma therapy.^10,31^ Our present studies support that the Syrian hamster can be used as an animal model for SARS-CoV-2 infection.

In conclusion, our study demonstrated the male Syrian hamster is more susceptible for SARS-CoV-2 infection to have lung pathogenesis than the females. Further studies on how SARS-CoV-2 causes more severe COVID-19 in males and whether inflammation is related to the gender-related outcomes will be carried out using the hamster models.

## Materials and methods

### Facility, ethics, and biosafety statement

All experiments with infectious SARS-CoV-2 were performed in the biosafety level 3 (BSL-3) and animal biosafety level 3 (ABSL-3) facilities in the State Key Laboratory of Emerging Infectious Diseases, School of Public Health, The University of Hong Kong. The animal studies were carried out in strict accordance with the recommendations in the Guide for the Care and Use of Laboratory Animals of the Ministry of Science and Technology of the People’s Republic of China. The protocols were approved by the Committee on the Ethics of the State Key Laboratory of Molecular Vaccinology and Molecular Diagnostics and the National Institute of Diagnostics and Vaccine Development in Infectious Diseases. Our staff wear powered air-purifying respirators that filter the air, and disposable coveralls when they culture the virus and handle animals that are in isolators. The researchers are disinfected before they leave the room and then shower on exiting the facility. All facilities, procedures, training records, safety drills, and inventory records are subject to periodic inspections and ongoing oversight by the institutional biosafety officers who consult frequently with the facility managers.

### Experimental animals

The Golden Syrian Hamster strain was derived from Charles River Laboratories and raised at the specific pathogen-free animal feeding facilities. All the animal experiments were approved by the Ethics Committee of the State Key Laboratory of Molecular Vaccinology and Molecular Diagnostics and the National Institute of Diagnostics and Vaccine Development in Infectious Diseases. Male and female hamsters of 8–14 weeks old were used for challenge of SARS-CoV-2 or controls.

### Virus stock

Viral stocks were prepared in Vero cells with DMEM containing 2% FBS, 5 μg/mL TPCK-trypsin, and 30 mmol/L MgCl_2_. Viruses were harvested and the titers were determined by means of plaque assay in Vero cells. Viral stocks (fourth-passage, 1.36 × 10^6^ PFU/mL, 1 mL per stock) were stored in −80 °C ultra-low-temperature refrigerator for animal study.

### Virus inoculation, sample collections, and observation of symptoms

The hamsters were anesthetized by isoflurane and nasally inoculated with the indicated dose of SARS-CoV-2 diluted in 100 μL PBS. For the study of gender-biases, male and female hamsters received a dose of 1 × 10^4^ PFU. To investigate the susceptibility to SARS-CoV-2, four groups of male hamsters received a dose gradient of 1 × 10^2^, 1 × 10^3^, 1 × 10^4^, and 1 × 10^5^ PFU, respectively. After SARS-CoV-2 infection, these hamsters were daily observed for illness symptoms include weakness, piloerection, hunched back, and abdominal respiration. Each symptom was scored based on the severity of none (0), moderate (1), mild (2), severe (3), and very severe (4), respectively. The videos of controls and hamsters with typical illness symptoms were recorded by a camera. Body weight of these hamsters was daily measured by electronic balance from 0 to 7 dpi. Nasal washings were daily collected for detection of viral RNA and titer. At 3, 5, and 7 dpi, hamsters were euthanized. Serum was collected for detection of SARS-CoV-2 RBD-specific IgG. The lung tissues were collected for gross observation and pathological analysis.

### Measurement of nasal washings and serum

To quantitate the viral RNA copies in the nasal washings, viral RNA was extracted by using a QIAamp vRNA mini-kit (Qiagen, Hilden, Germany) according to the manufacturer’s instructions. Real-time quantitative PCR was conducted by using the SLAN-96S Real-Time System (Hongshi, Shanghai, China) with a COVID-19 RT-PCR Kit from Wantai (Beijing, China). Relative Viral RNA of SARS-CoV-2 N protein was determined using primer pairs and probes shown in the kit instruction. Viral RNA copies were expressed on a log_10_ scale after normalized to the standard curve obtained by using ten-fold dilutions of a SARS-CoV-2 stock with known viral titer. Titer of live virus in nasal washings was measured by standard TCID_50_ method or plaque assay in Vero cells. SARS-CoV-2 RBD-specific IgG in hamster serum was detected by using a double-antigen sandwich ELISA Kit (Wantai, Beijing, China).

### Pathological analysis

For pathological analysis, lung tissues were fixed in formalin for 48 h, dehydrated, and then embedded in paraffin wax. The wax block of lung tissues was cut into 4-μm sections for several pathological staining and analysis. H&E staining was employed for analysis of general lung pathogenic lesions include pulmonary edema, consolidation, and inflammation. The standards for pathological score of lung tissues in this study are derived from a recent study of SARS-CoV-2 infection in hamster model.^10^ In brief, H&E staining result of each lung lobe was analyzed for its severity of pathological change. The pathological score includes (a) Alveolar septum thickening and consolidation; (b) Hemorrhage, exudation, pulmonary edema, and mucous; (c) Recruitment and infiltration of inflammatory immune cells. For each issue, score related to the severity: 0 indicates no pathological change was observed, 1 indicates moderate pathological change, 2 indicates mild pathological change, 3 indicates severe pathological change, and 4 indicates very severe pathological change. In conclusion, scores of such three issues were added as the comprehensive lung pathological score of a lung lobe. Masson staining was employed for analysis of lung fibrosis. Immunohistochemistry staining for SARS-CoV-2 nucleocapsid protein (NP) was employed for analysis of viral antigen expression and distribution in lung tissues. A mouse anti-SARS-CoV-2 N protein-specific antibody (15A7-1, house-keeping) was used as the first antibody of immunohistochemistry staining. The pathological reagents include immunohistochemistry Kits (#KIT-9730), Masson staining Kits (#MST-8004), Hematoxylin (#CTS-1096), and Eosin (#CTS-4094) were purchased from Maxim Biotechnology (Fuzhou, China). The images of whole lung lobes were screened by a high-throughput screening microscope system (EVOS M7000, Invitrogen of Thermo Fisher Scientific). The high magnification images were taken by a high-resolution microscope (AXIO Imagier.A2, Zeiss). Comprehensive pathological score of lung sections was performed according to the degree of lung lesions include alveolar septum hyperplasia, consolidation and impairment of alveolar structure, fluid exudation, mucus suppository, thrombus, inflammation recruitment, and infiltration of immune cells in each individual lung lobes.

### Statistical analysis

Student’s unpaired two-tailed *t*-test and one-way ANOVA were performed using GraphPad Prism 8.0 (GraphPad Software). Data are presented as the means ± SD. Two-sided *p*-values <0.05 were considered significant: *P < 0.05, **P < 0.01, ***P < 0.001, *NS* indicates no significance.

## Supplementary information

Supplementary Materials

Video 1

Video 2

Video 3

## Data Availability

All data collected in this study are available from the corresponding authors upon reasonable request.
