# Supplementary material for: Gender associates with both susceptibility to infection and pathogenesis of SARS-CoV-2 in Syrian hamster
Source: Signal Transduct Target Ther. 2021 Mar 31;6:136. doi: 10.1038/s41392-021-00552-0 (PMC8009924; doi:10.1038/s41392-021-00552-0)
Supplement: Supplementary file 1 — Supplementary Materials [file 41392_2021_552_MOESM1_ESM.pdf]

Supplementary Materials for

Gender associates with both susceptibility to infection and pathogenesis of SARS-CoV-2 in Syrian hamster

Lunzhi Yuan[1], Huachen Zhu[2,3], Ming Zhou[1], Jian Ma[1], Rirong Chen[2,3], Yao Chen[1], Liqiang Chen[2,3], Kun Wu[1], Minping Cai[2,3], Junping Hong[1], Lifeng Li[2,3], Che Liu[1], Huan Yu[2,3], Yali Zhang[1], Jia Wang[2,3], Tianying Zhang[1], Shengxiang Ge[1], Jun Zhang[1], Quan Yuan[1], Yixin Chen[1], Qiyi Tang[4], Honglin Chen[2,3], Tong Cheng[1], Yi Guan[2,3] and Ningshao Xia[1,5]

Correspondence to: Tong Cheng (tcheng@xmu.edu.cn); Yi Guan (yguan@hku.hk); Ningshao Xia (nsxia@xmu.edu.cn)

This PDF file includes:

Figures. S1 to S19

Tables S1 to S3

Captions for Movies V1 to V3

Other Supplementary Materials for this manuscript include the following:

Movies V1 to V3

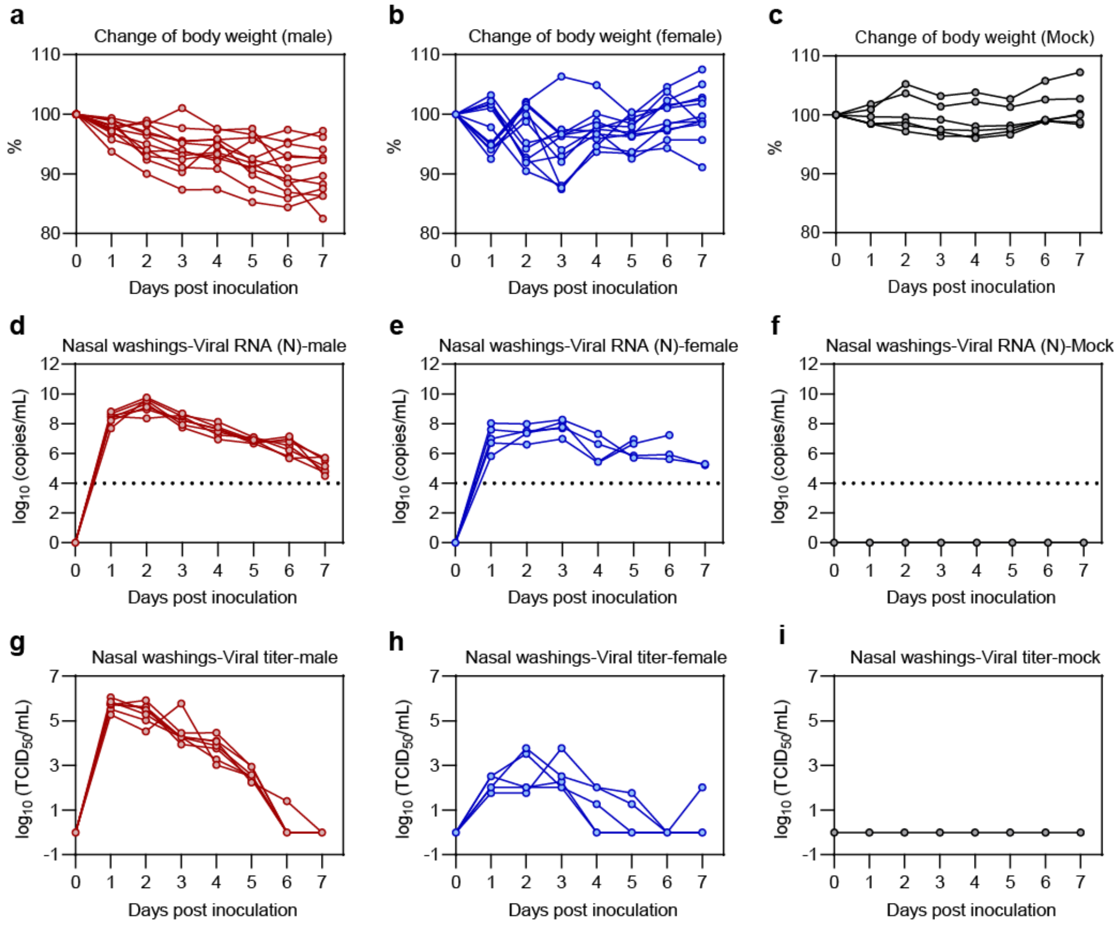

Supplementary Fig. 1. Body weight change of individual **(a)** male and **(b)** female hamsters infected with SARS-CoV-2, and the **(c)** mock animals. Viral RNA in nasal washings of individual **(d)** male and **(e)** female hamsters infected with SARS-CoV-2, and the **(f)** mock animals. Viral titer in nasal washings of individual **(g)** male and **(h)** female hamsters infected with SARS-CoV-2, and the **(i)** mock animals.

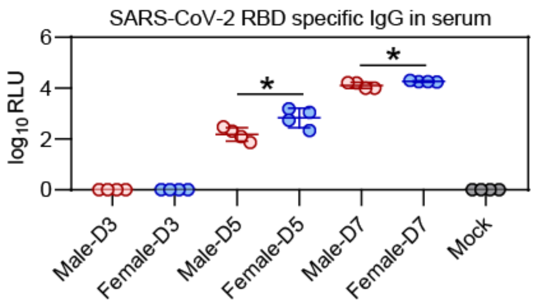

Supplementary Fig. 2. Serum SARS-CoV-2 RBD specific IgG levels of male and female hamsters (n=4/group).

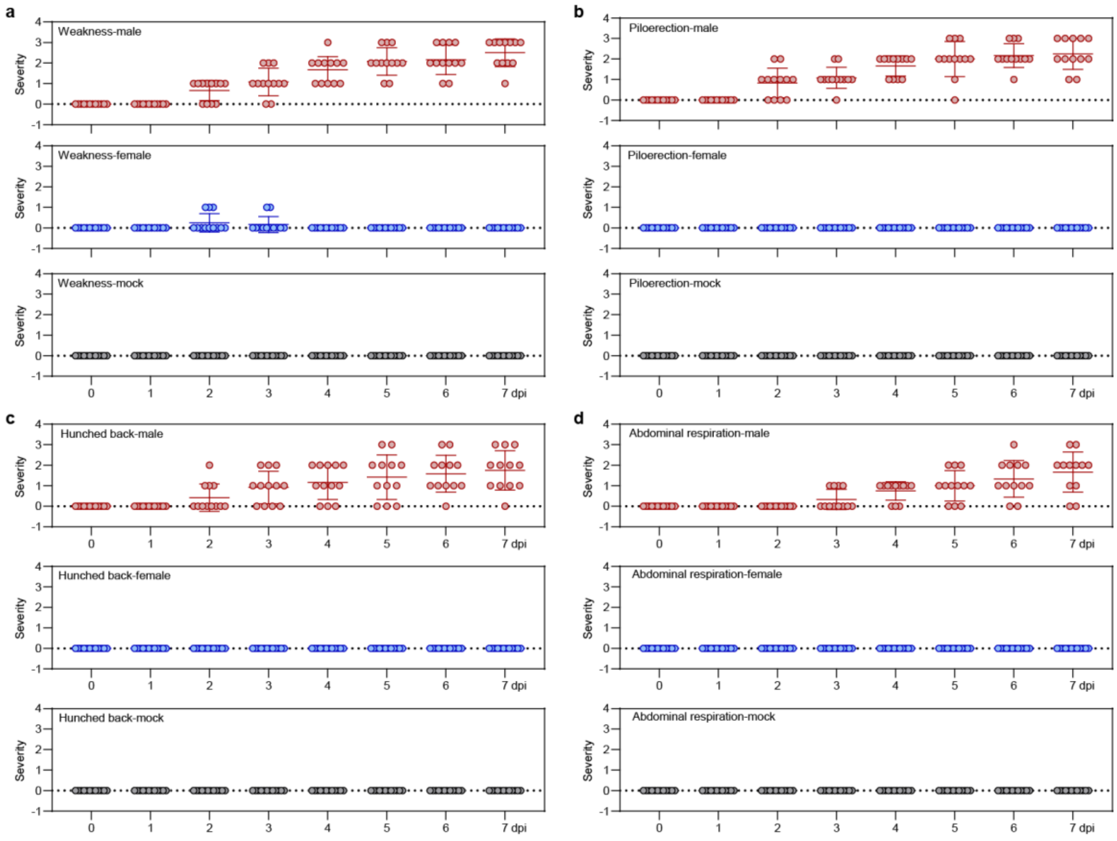

Supplementary Fig. 3. Symptom scores include **(a)** Weakness, **(b)** Piloerection, **(c)** Hunched back and **(d)** Abdominal respiration of individual male and female hamsters and the mock animals.

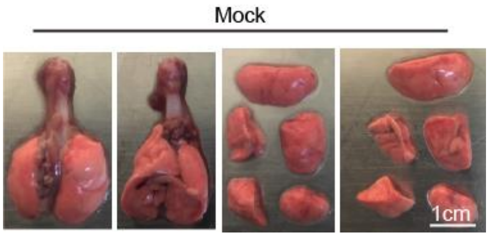

Supplementary Fig. 4. Gross lung lesions of a mock animal.

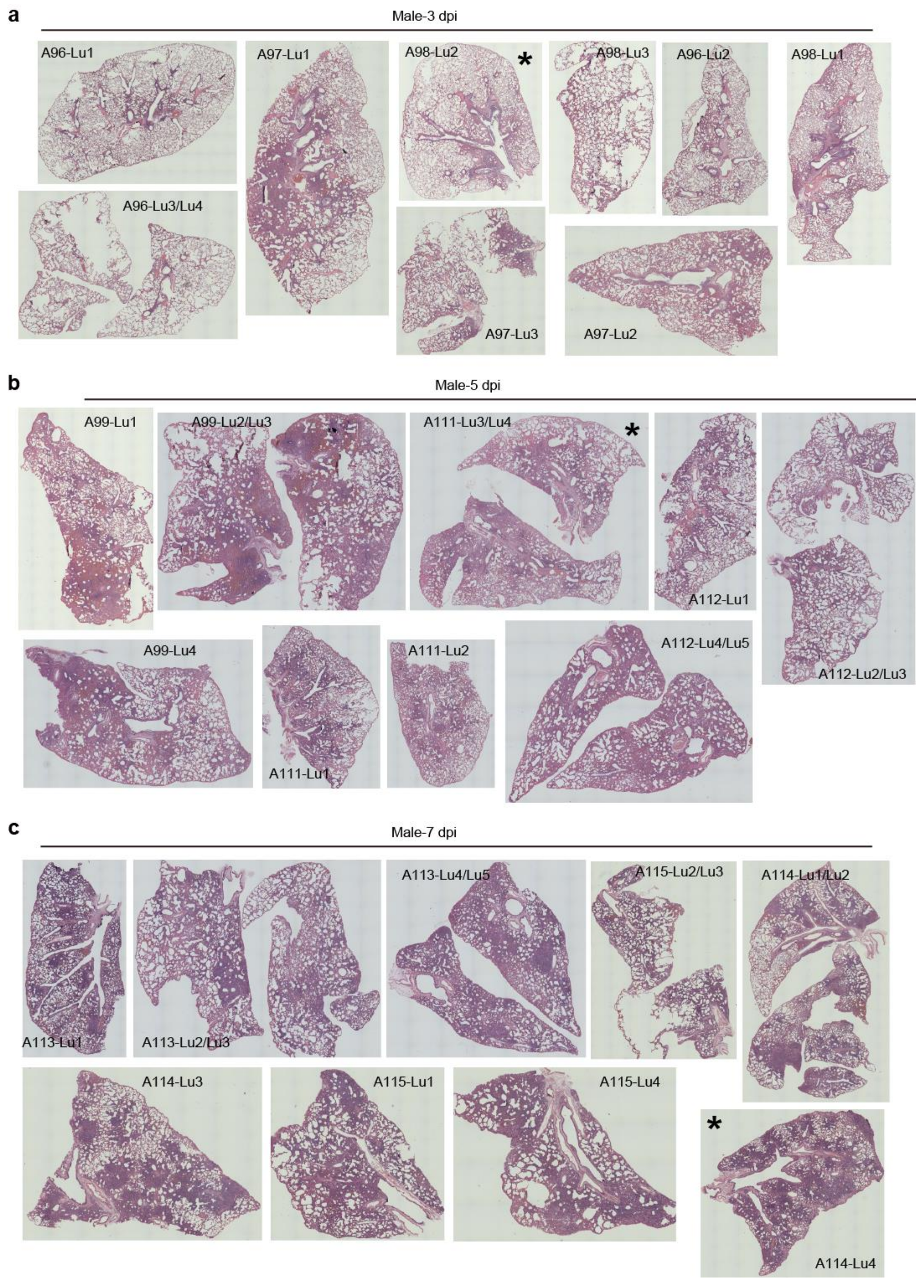

Supplementary Fig. 5. H&E staining for lung lobes collected from individual male hamsters inoculated with 1×10^4 PFU of SARS-CoV-2 at **(a)** 3 dpi, **(b)** 5 dpi and **(c)** 7 dpi, respectively. The images of A98-Lu2, A111-Lu2/4 and A114-Lu4 marked by asterisk were representative lung lobes shown in Fig.2e.

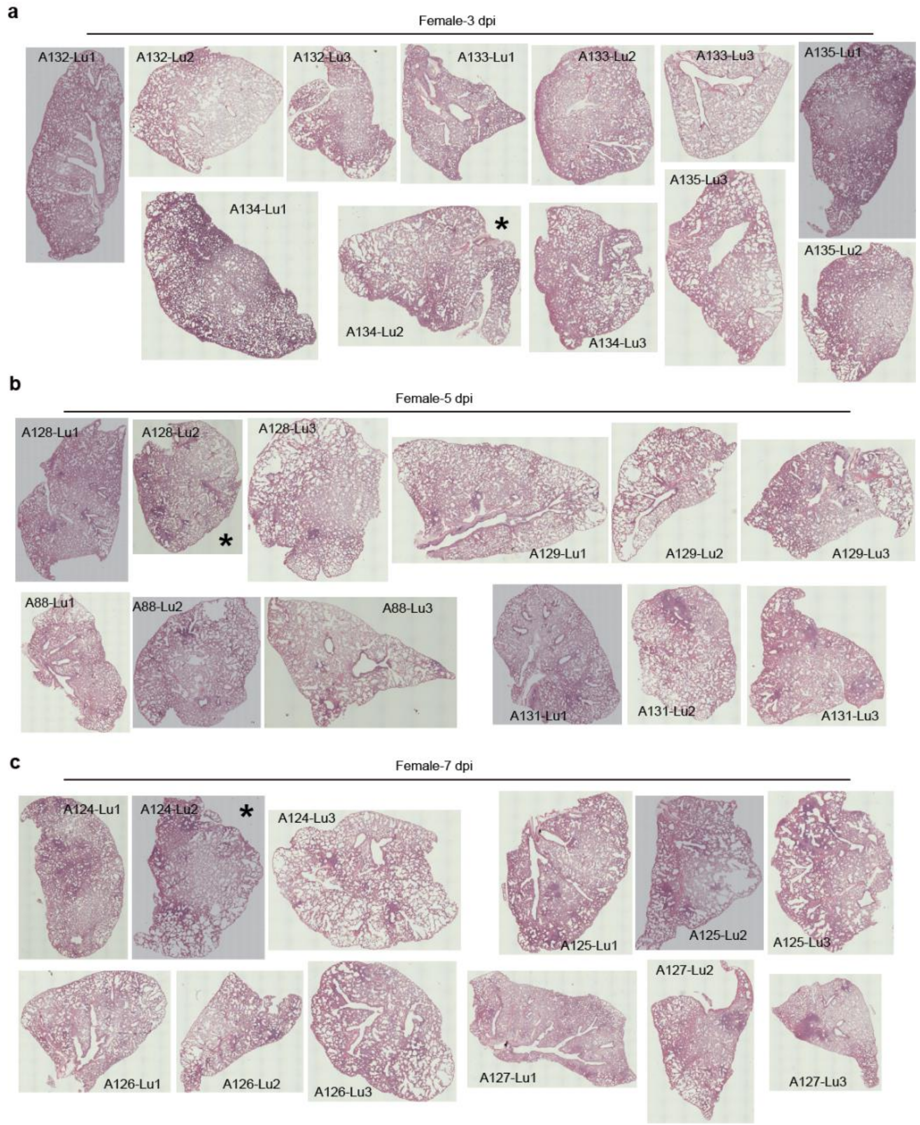

Supplementary Fig. 6. H&E staining for lung lobes collected from individual female hamsters inoculated with 1×10^4 PFU of SARS-CoV-2 at **(a)** 3 dpi, **(b)** 5 dpi and **(c)** 7 dpi, respectively. The images of A134-Lu2, A128-Lu2 and A124-Lu2 marked by asterisk were representative lung lobes shown in Fig.2f.

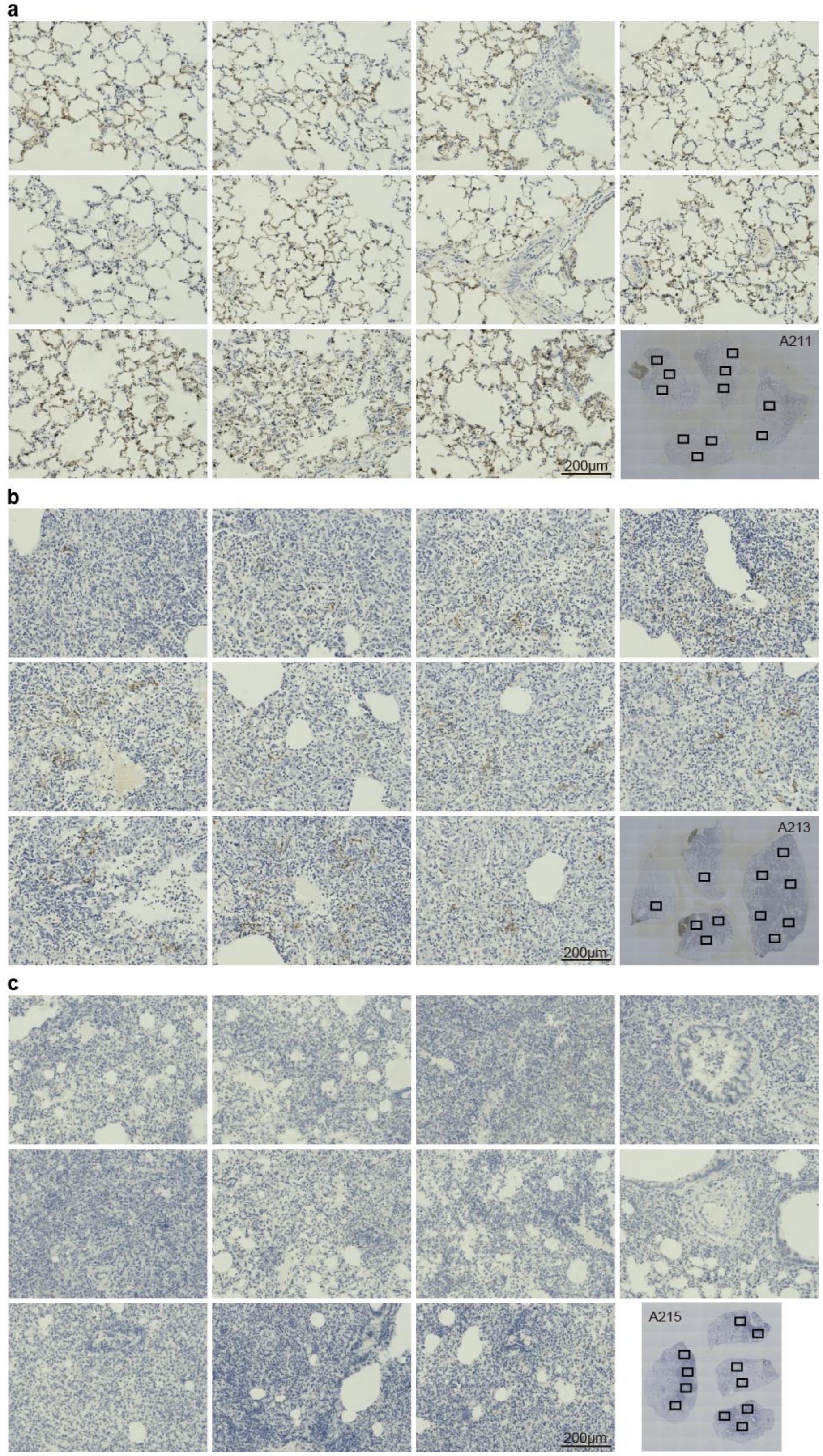

Supplementary Fig. 7. Immunohistochemistry staining for expression of SARS-CoV-2 N protein in lung lobes collected from male hamsters inoculated with 10^4 PFU of SARS-CoV-2 at **(a)** 3 dpi, **(b)** 5 dpi and **(c)** 7 dpi, respectively. As shown, images were randomly taken from different views in the lung lobes.

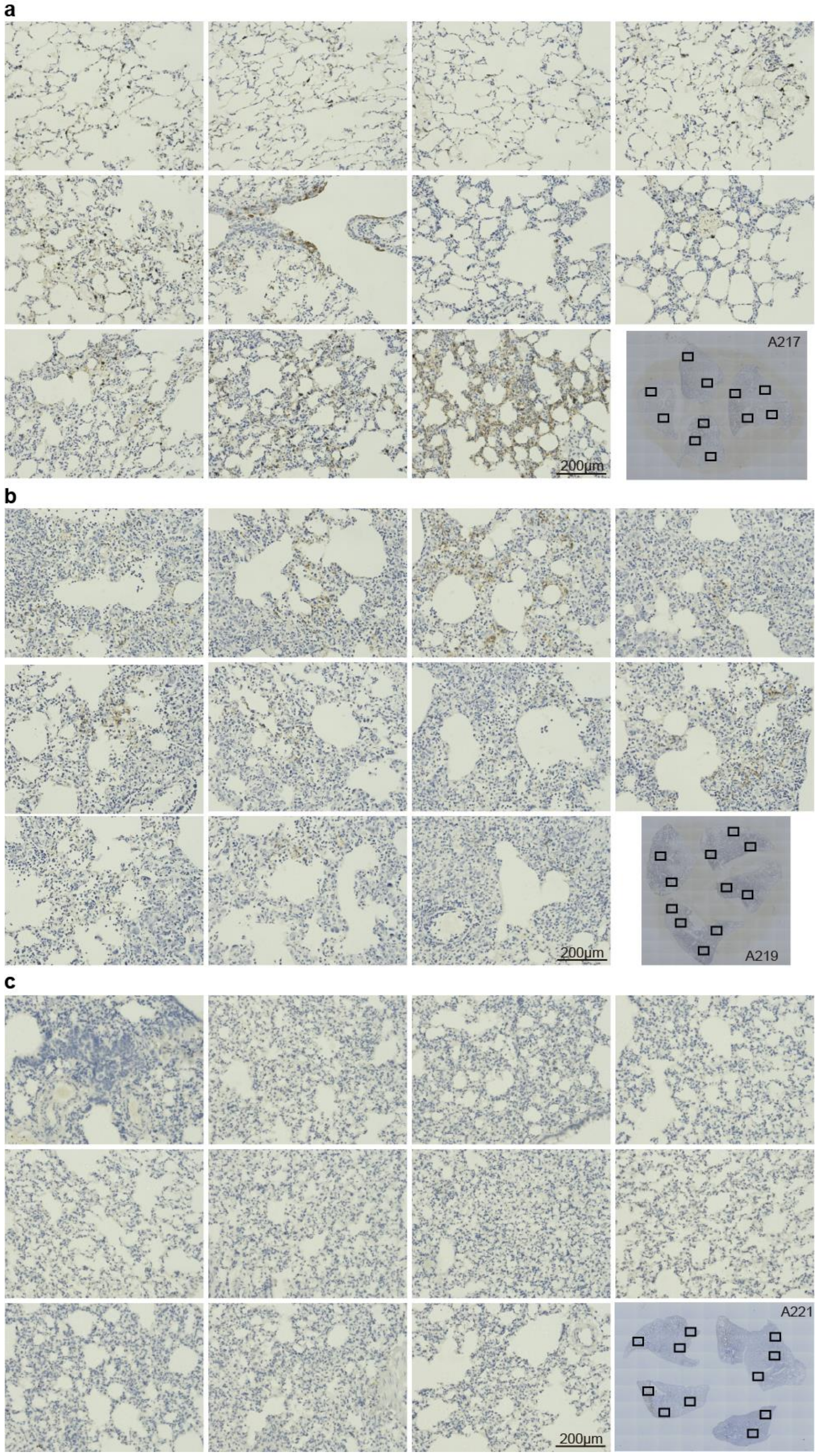

Supplementary Fig. 8. Immunohistochemistry staining for expression of SARS-CoV-2 N protein in lung lobes collected from female hamsters inoculated with 10^4 PFU of SARS-CoV-2 at **(a)** 3 dpi, **(b)** 5 dpi and **(c)** 7 dpi, respectively. As shown, images were randomly taken from different views in the lung lobes.

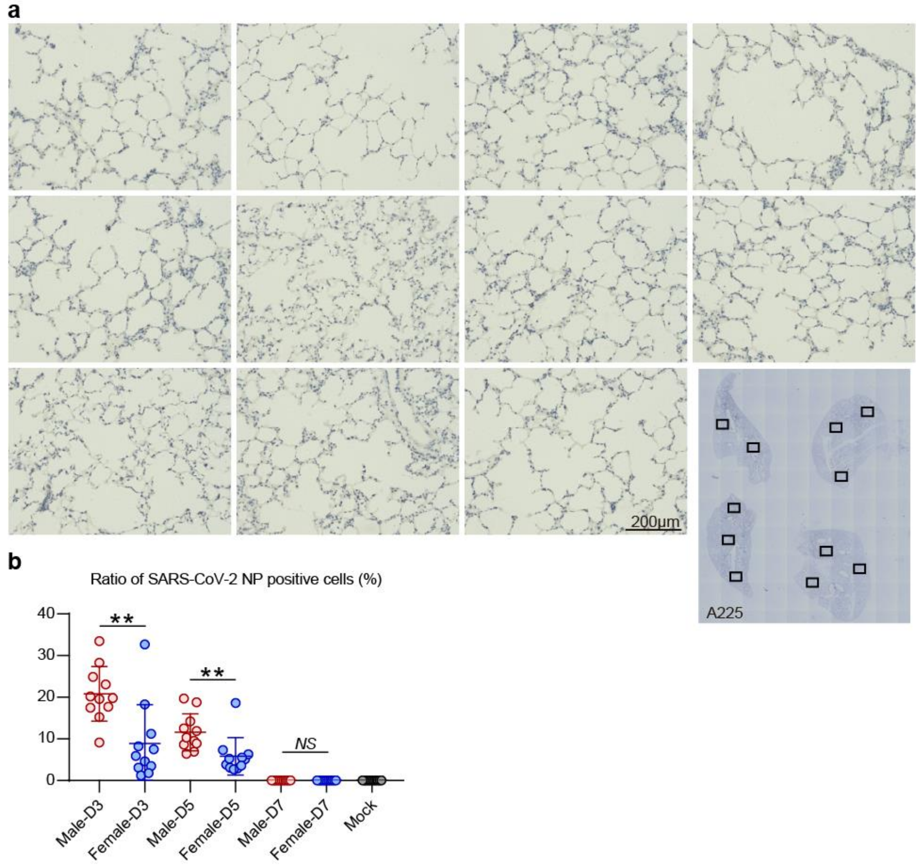

Supplementary Fig. 9. (a) Immunohistochemistry staining for expression of SARS-CoV-2 N protein in lung lobes collected from a mock hamster without SARS-CoV-2 infection. As shown, images were randomly taken from different views in the lung lobes. **(b)** Statically analysis for the ratio of SARS-CoV-2 NP positive cells in lung tissues collected from male and female hamsters inoculated with 10^4 PFU of SARS-CoV-2, and the mock hamster without infection.

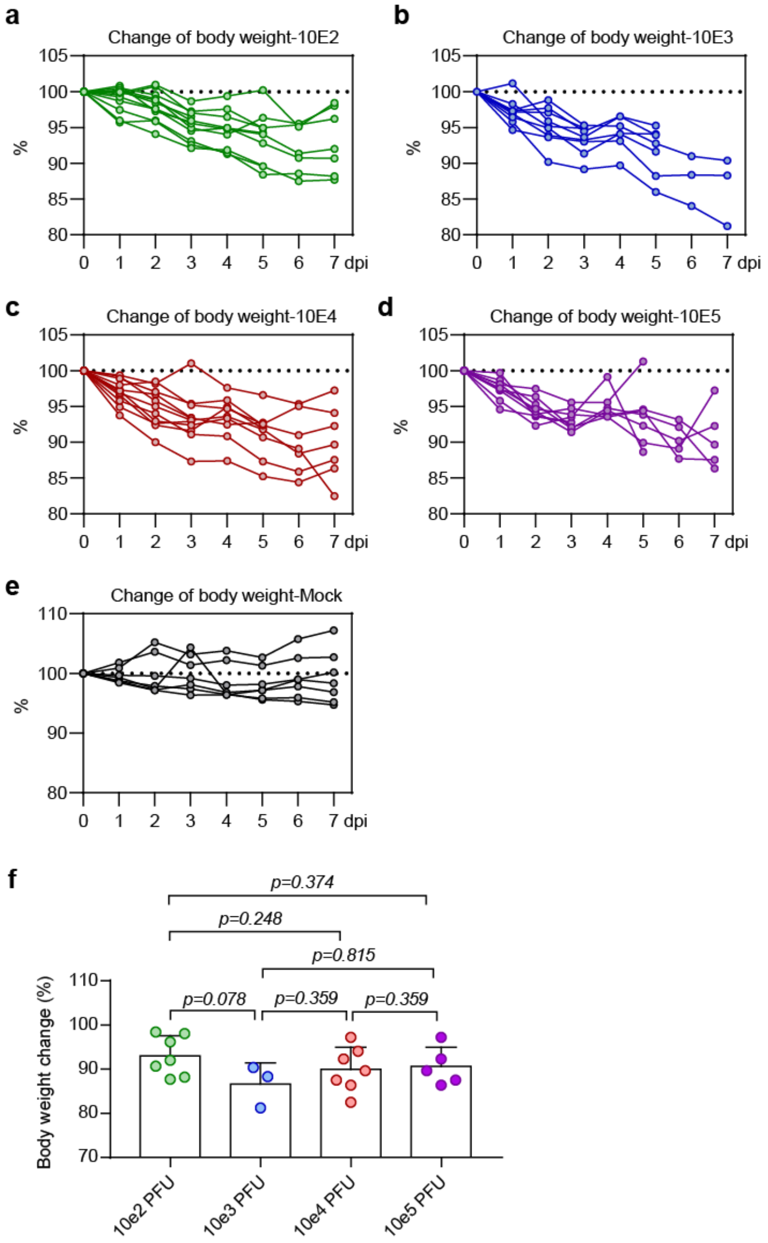

Supplementary Fig. 10. Body weight change of individual male hamsters challenged with a dose gradient from 1×10^2 to 1×10^5 PFU of SARS-CoV-2. **(a)** Data of 12 hamsters with 1×10^2 PFU of SARS-CoV-2 infection were collected from 0 to 3 dpi, data of 10 hamsters were collected from 4 to 5 dpi and data of 7 hamsters were collected from 6 to 7 dpi. **(b)** Data of 9 hamsters with 1×10^3 PFU of SARS-CoV-2 infection were collected from 0 to 3 dpi, data of 7 hamsters were collected from 4 to 5 dpi and data of 3 hamsters were collected from 6 to 7 dpi. **(c)** Data of 11 hamsters with 1×10^4 PFU of SARS-CoV-2 infection were collected from 0 to 3 dpi, data of 9 hamsters were collected from 4 to 5 dpi and data of 7 hamsters were collected from 6 to 7 dpi. **(d)** Data of 9 hamsters with 1×10^5 PFU of SARS-CoV-2 infection were collected from 0 to 3 dpi, data of 7 hamsters were observed from 4 to 5 dpi and data of 5 hamsters were observed

from 6 to 7 dpi. **(e)** Data of 7 hamsters without infection were collected from 0 to 7 dpi. **(f)** Body weight loss of male hamsters inoculated with 1×10^2 to 1×10^5 PFU of SARS-CoV-2 showed no significant difference at 7 dpi (unpaired t test was performed between the groups).

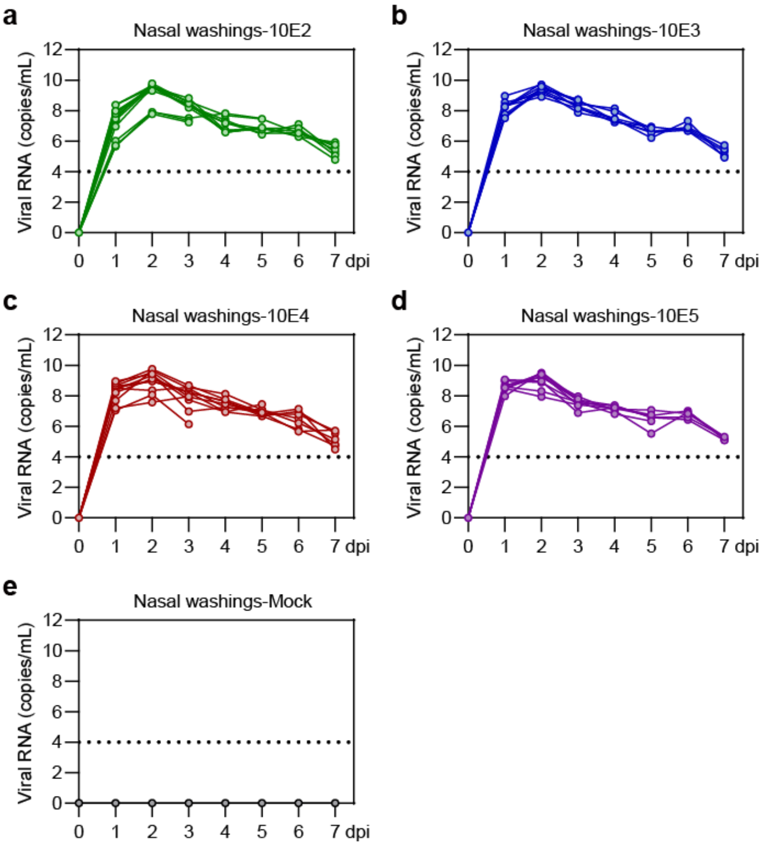

Supplementary Fig. 11. Viral RNA load in nasal washings of individual male hamsters challenged with a dose gradient from 1×10^2 to 1×10^5 PFU of SARS-CoV-2. Data of 12 hamsters with 1×10^2 PFU of SARS-CoV-2 infection were collected from 0 to 3 dpi, data of 10 hamsters were collected from 4 to 5 dpi and data of 7 hamsters were collected from 6 to 7 dpi; data of 9 hamsters with 1×10^3 PFU of SARS-CoV-2 infection were collected from 0 to 3 dpi, data of 7 hamsters were collected from 4 to 5 dpi and data of 5 hamsters were collected from 6 to 7 dpi; data of 11 hamsters with 1×10^4 PFU of SARS-CoV-2 infection were collected from 0 to 3 dpi, data of 9 hamsters were collected from 4 to 5 dpi and data of 7 hamsters were collected from 6 to 7 dpi; data of 9 hamsters with 1×10^5 PFU of SARS-CoV-2 infection were collected from 0 to 3 dpi, data of 7 hamsters were observed from 4 to 5 dpi and data of 5 hamsters were observed from 6 to 7 dpi; data of 7 hamsters without infection were collected from 0 to 7 dpi.

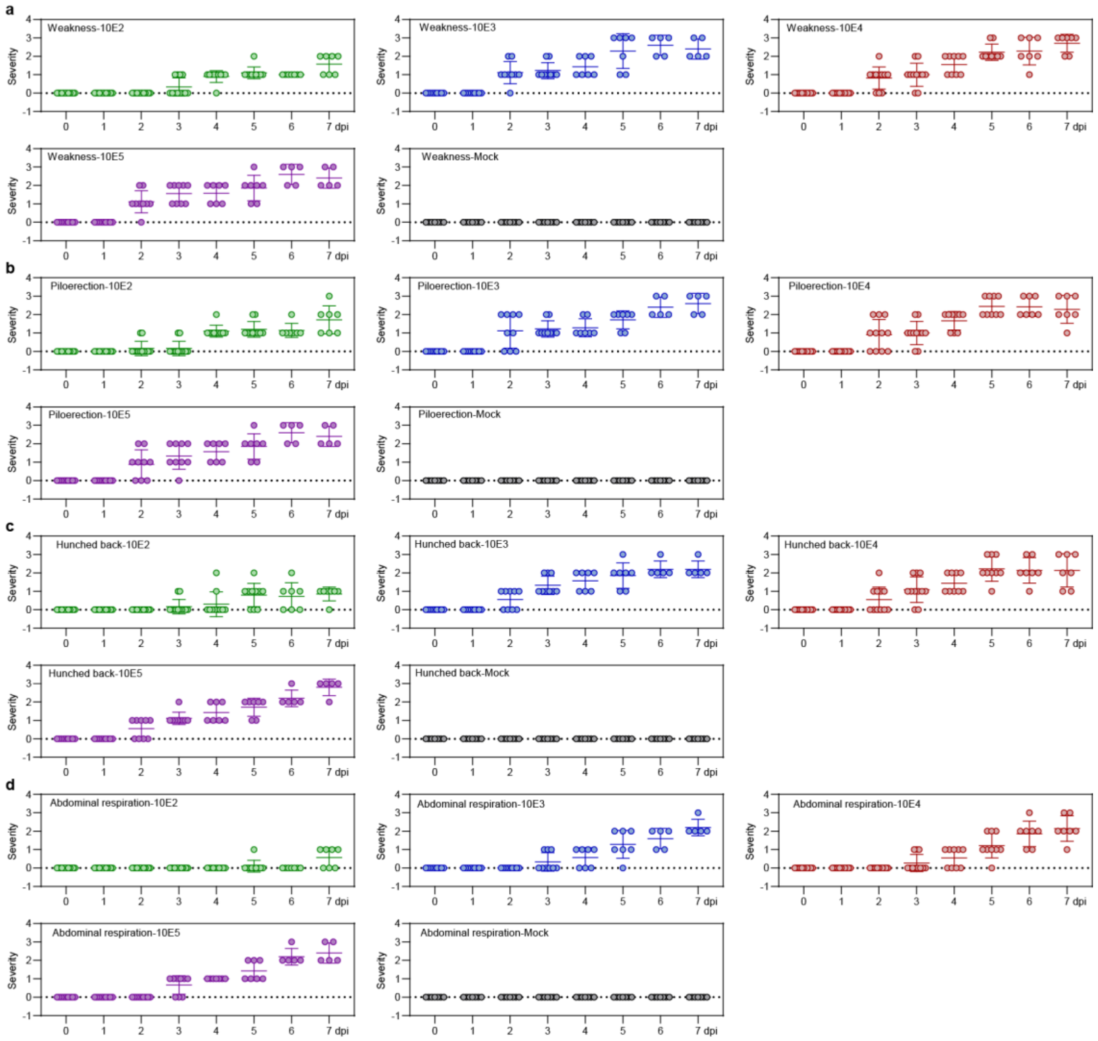

Supplementary Fig. 12. Symptom scores of individual male hamsters challenged with a dose gradient from $1{\times}10^2$ to $1{\times}10^5$ PFU of SARS-CoV-2. Symptoms of 12 hamsters with $1{\times}10^2$ PFU of SARS-CoV-2 infection were observed from 0 to 3 dpi, 10 of such hamsters were observed from 4 to 5 dpi and 7 of such hamsters were observed from 6 to 7 dpi; symptoms of 9 hamsters with $1{\times}10^3$ PFU of SARS-CoV-2 infection were observed from 0 to 3 dpi, 7 of such hamsters were observed from 4 to 5 dpi and 5 of such hamsters were observed from 6 to 7 dpi; symptoms of 11 hamsters with $1{\times}10^4$ PFU of SARS-CoV-2 infection were observed from 0 to 3 dpi, 9 of such hamsters were observed from 4 to 5 dpi and 7 of such hamsters were observed from 6 to 7 dpi; symptoms of 9 hamsters with $1{\times}10^5$ PFU of SARS-CoV-2 infection were observed from 0 to 3 dpi, 7 of such hamsters were observed from 4 to 5 dpi and 5 of such hamsters were observed from 6 to 7 dpi; symptoms of 7 hamsters without infection were observed from 0 to 7 dpi.

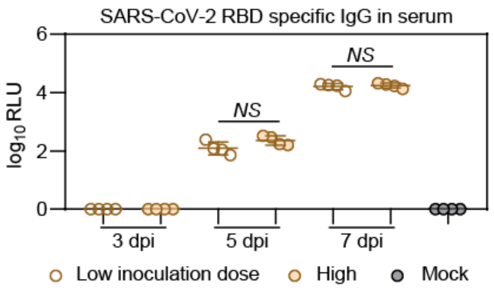

Supplementary Fig. 13. The levels of SARS-CoV-2 RBD specific IgG in serum of male hamsters that inoculated with low dose (1×10^2 and 1×10^3 PFU) and high dose (1×10^4 and 1×10^5 PFU) of SARS-CoV-2 at 3, 5 and 7 dpi were measured by ELISA, respectively (n=4/group).

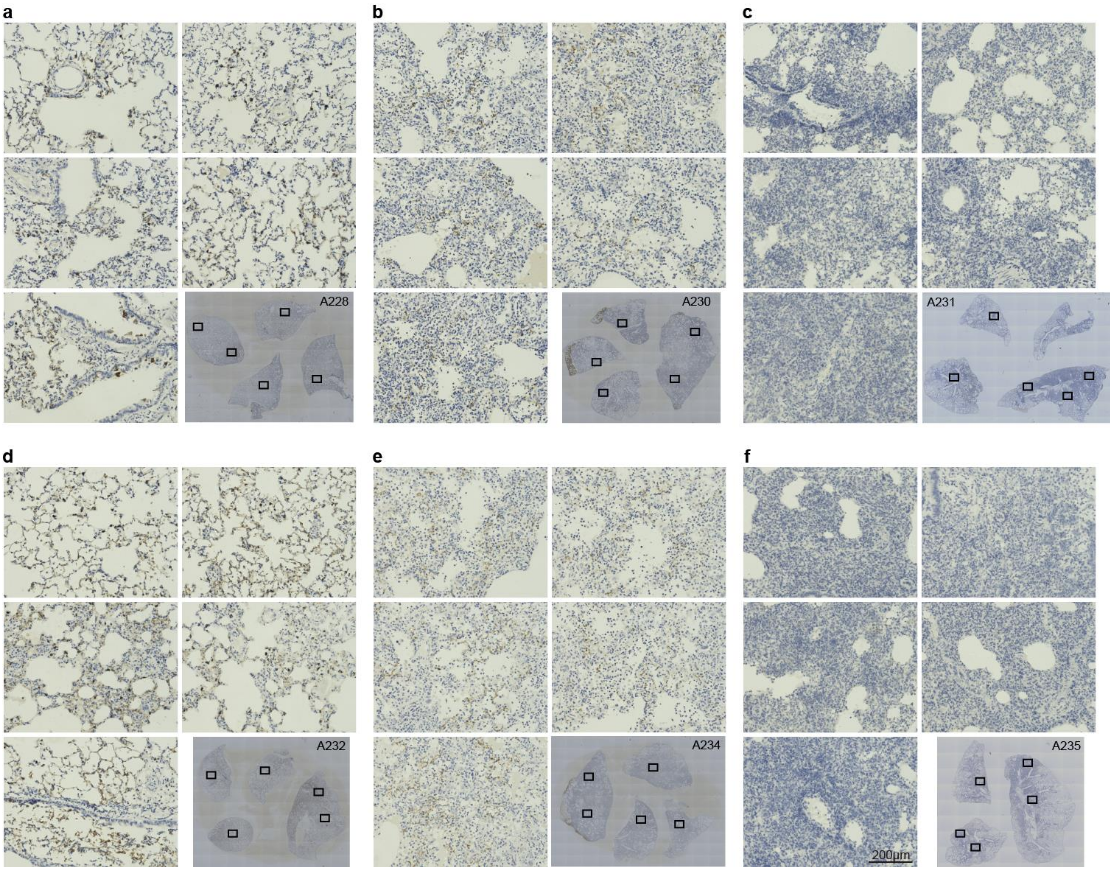

Supplementary Fig. 14. Immunohistochemistry staining for expression of SARS-CoV-2 N protein in lung sections collected from male hamsters challenged with a dose gradient from 1×10^2 PFU of SARS-CoV-2 at **(a)** 3 dpi, **(b)** 5 dpi and **(c)** 7 dpi, respectively; male hamsters challenged with a dose gradient from 1×10^3 PFU of SARS-CoV-2 at **(d)** 3 dpi, **(e)** 5 dpi and **(f)** 7 dpi, respectively. Images were randomly taken from different views in the lung lobes (bar=200μm).

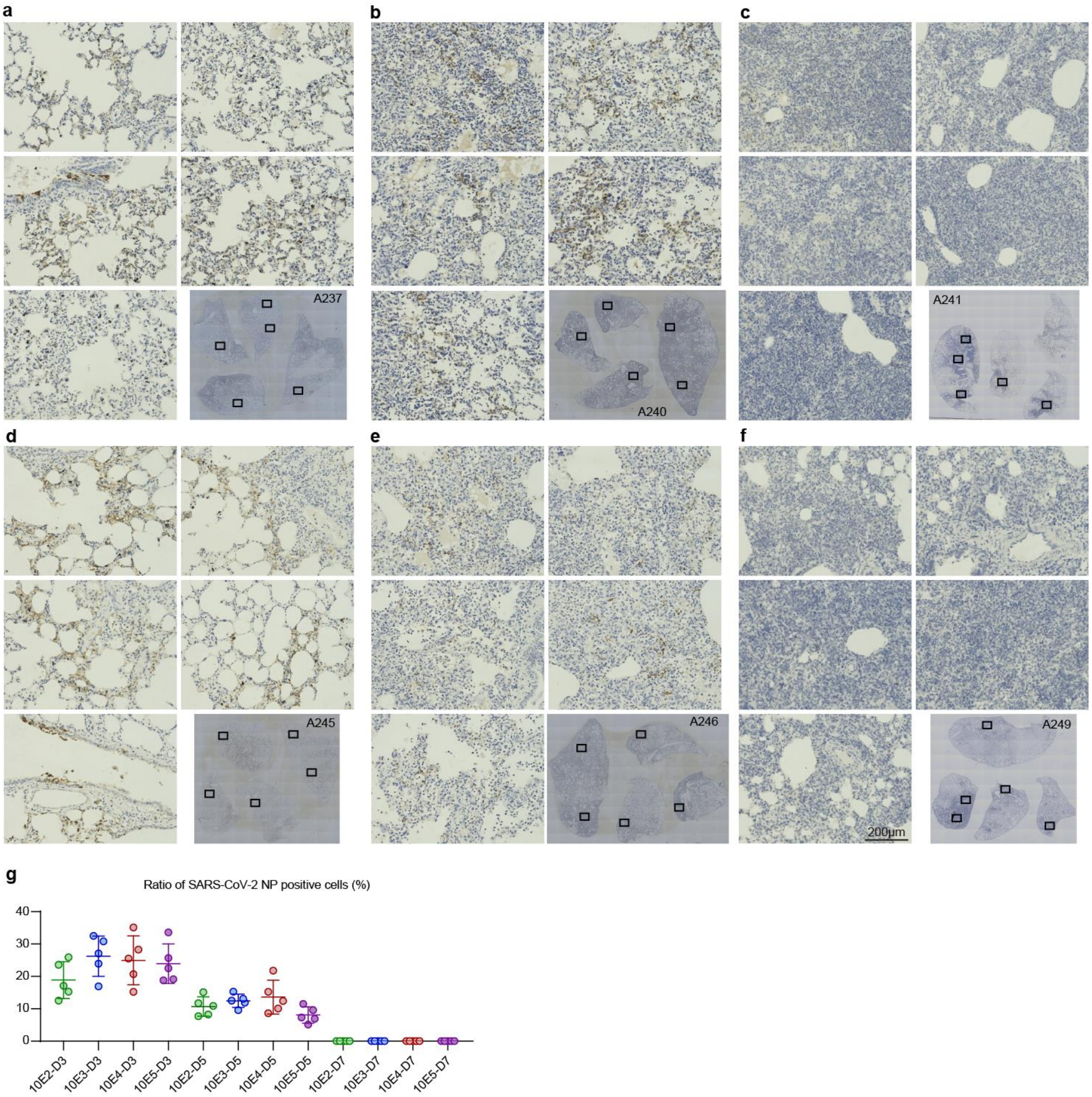

Supplementary Fig. 15. Immunohistochemistry staining for expression of SARS-CoV-2 N protein in lung sections collected from male hamsters challenged with a dose gradient from 1×10^4 PFU of SARS-CoV-2 at **(a)** 3 dpi, **(b)** 5 dpi and **(c)** 7 dpi, respectively; male hamsters challenged with a dose gradient from 1×10^5 PFU of SARS-CoV-2 at **(d)** 3 dpi, **(e)** 5 dpi and **(f)** 7 dpi, respectively. Images were randomly taken from different views in the lung lobes (bar=200μm). **(g)** Statically analysis for the ratio of SARS-CoV-2 NP positive cells in lung tissues collected from male and female hamsters inoculated with 1×10^4 PFU of SARS-CoV-2.

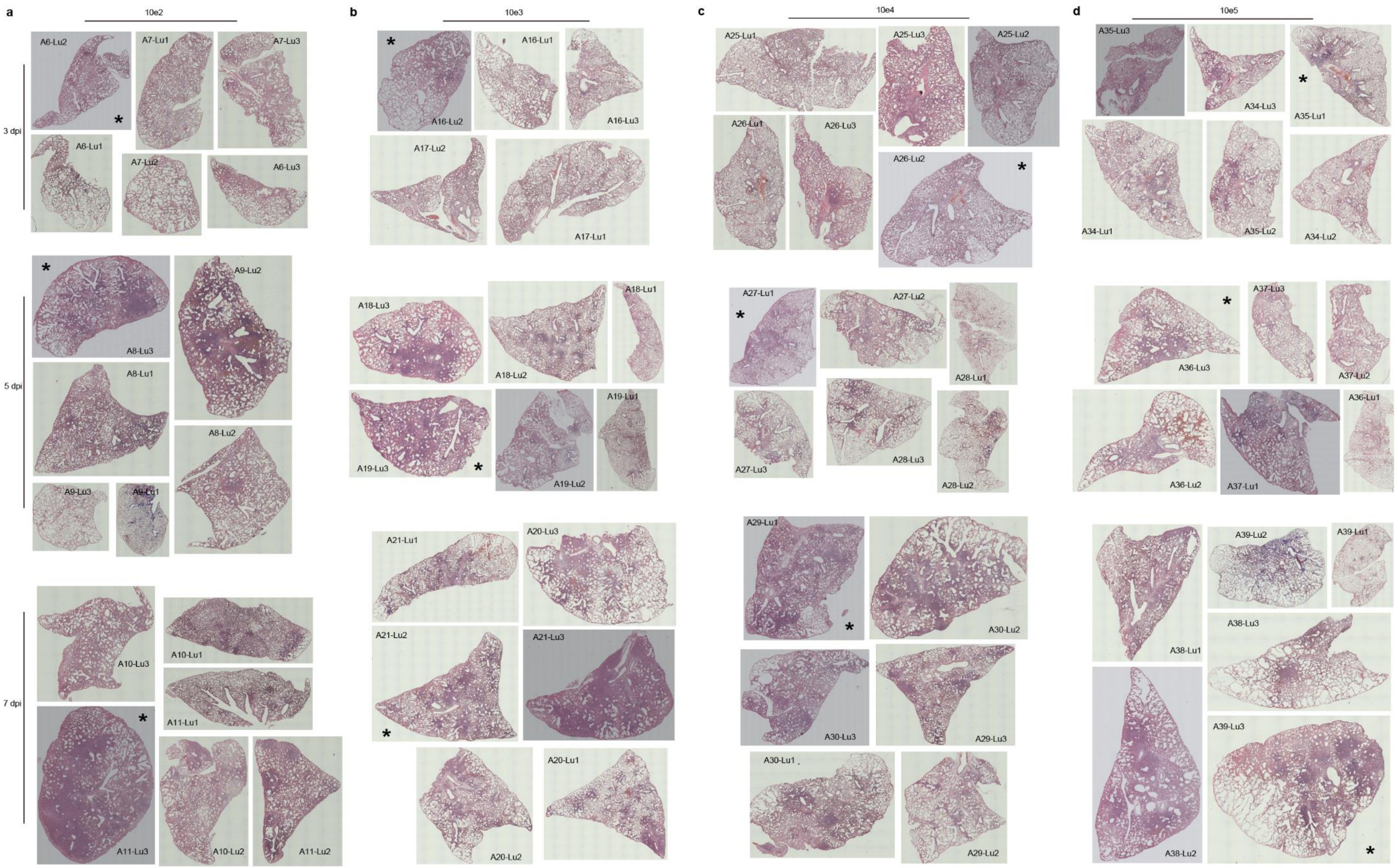
a
10e2
3 dpi
5 dpi
7 dpi
A6-Lu2
A7-Lu1
A7-Lu3
A6-Lu1
A7-Lu2
A6-Lu3
A8-Lu3
A9-Lu2
A8-Lu1
A8-Lu2
A9-Lu3
A9-Lu1
A10-Lu3
A10-Lu1
A11-Lu1
A11-Lu3
A10-Lu2
A11-Lu2
b
10e3
A16-Lu1
A16-Lu2
A16-Lu3
A17-Lu2
A17-Lu1
A18-Lu3
A18-Lu2
A18-Lu1
A19-Lu3
A19-Lu2
A19-Lu1
A21-Lu1
A20-Lu3
A21-Lu2
A21-Lu3
A20-Lu2
A20-Lu1
c
10e4
A25-Lu1
A25-Lu3
A25-Lu2
A26-Lu1
A26-Lu3
A26-Lu2
A27-Lu1
A27-Lu2
A28-Lu1
A27-Lu3
A28-Lu3
A28-Lu2
A29-Lu1
A30-Lu2
A30-Lu3
A29-Lu3
A30-Lu1
A29-Lu2
d
10e5
A35-Lu3
A34-Lu3
A35-Lu1
A34-Lu1
A35-Lu2
A34-Lu2
A37-Lu3
A36-Lu3
A37-Lu2
A36-Lu1
A36-Lu2
A37-Lu1
A39-Lu2
A39-Lu1
A38-Lu1
A38-Lu3
A39-Lu3
A38-Lu2

Supplementary Fig. 16. H&E staining for lung sections collected from individual male hamsters challenged with a dose gradient of **(a)** 1×10^2, **(b)** 1×10^3, **(c)** 1×10^4, and **(d)** 1×10^5 PFU of SARS-CoV-2 at 3 dpi, 5 dpi and 7 dpi respectively. The images of A6-Lu2, A8-Lu3, A11-Lu3, A16-Lu2, A19-Lu3, A21-Lu2, A26-Lu2, A27-Lu1, A29-Lu1, A35-Lu1, A36-Lu3 and A39-Lu3 and A124-Lu2 marked by asterisk were representative lung lobes shown in Fig.4.

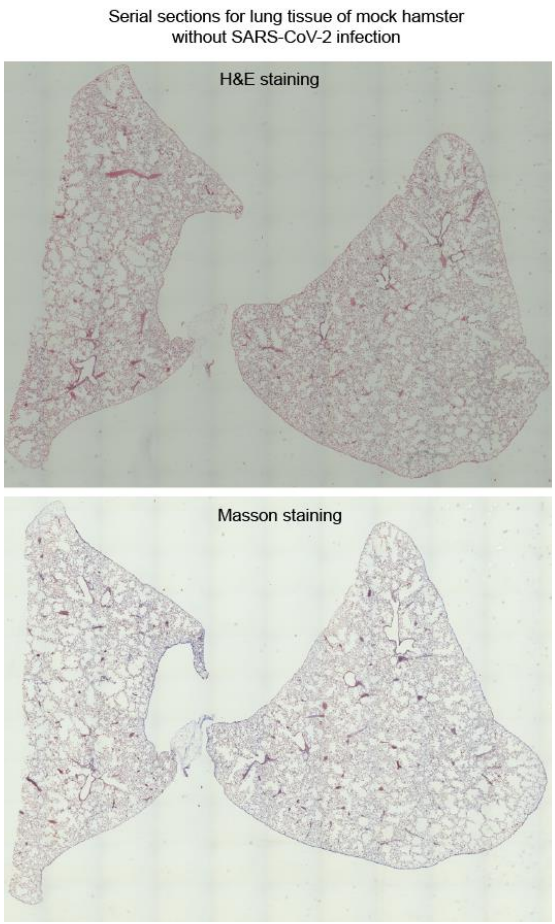

Supplementary Fig. 17. H&E staining and Masson staining of mock hamster.

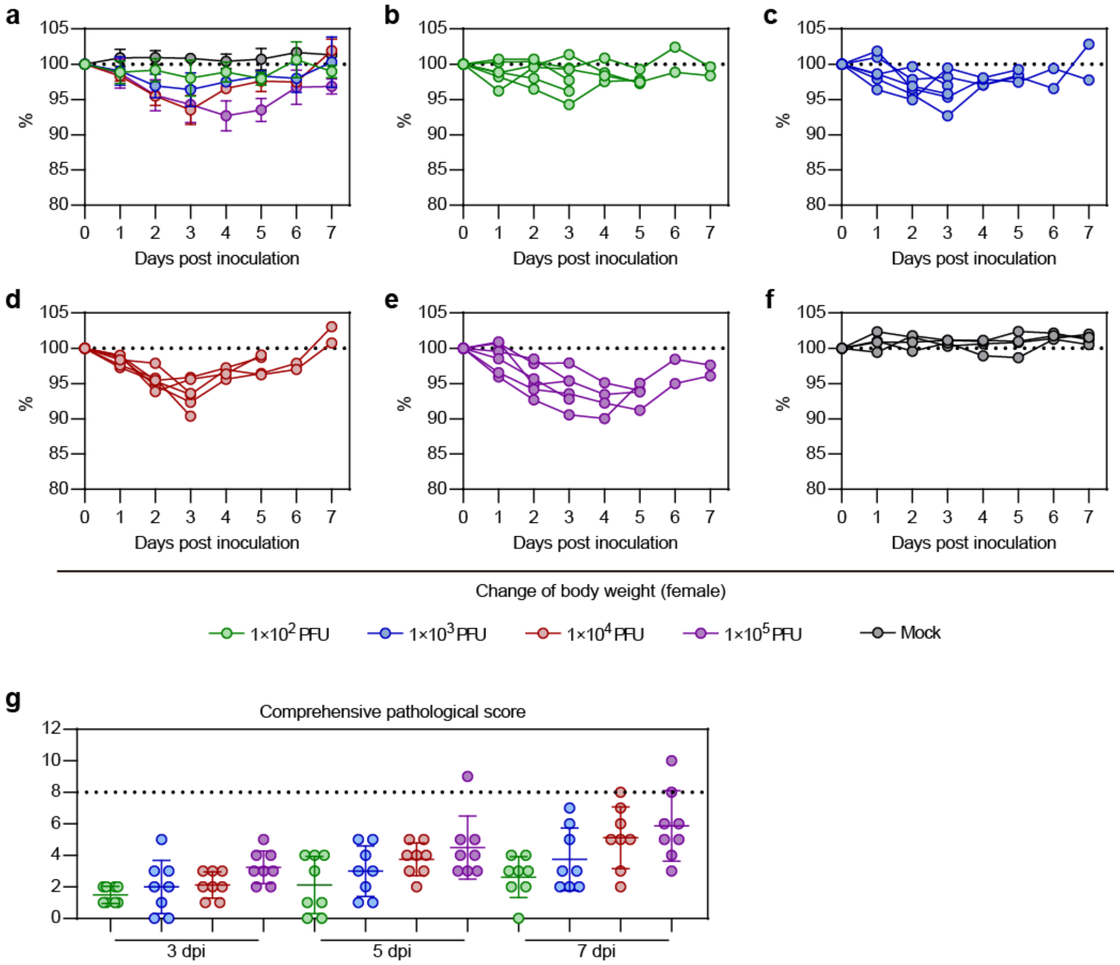

Supplementary Fig. 18. Body weight changes of **(a)** grouped and individual female hamsters challenged with a dose gradient of **(b)** 1×10^2, **(c)** 1×10^3, **(d)** 1×10^4, and **(e)** 1×10^5 PFU of SARS-CoV-2, and **(f)** the mock animals without infection, respectively. **(g)** Comprehensive pathological score of lung tissues collected these female hamsters (n=8 lung lobes/group).

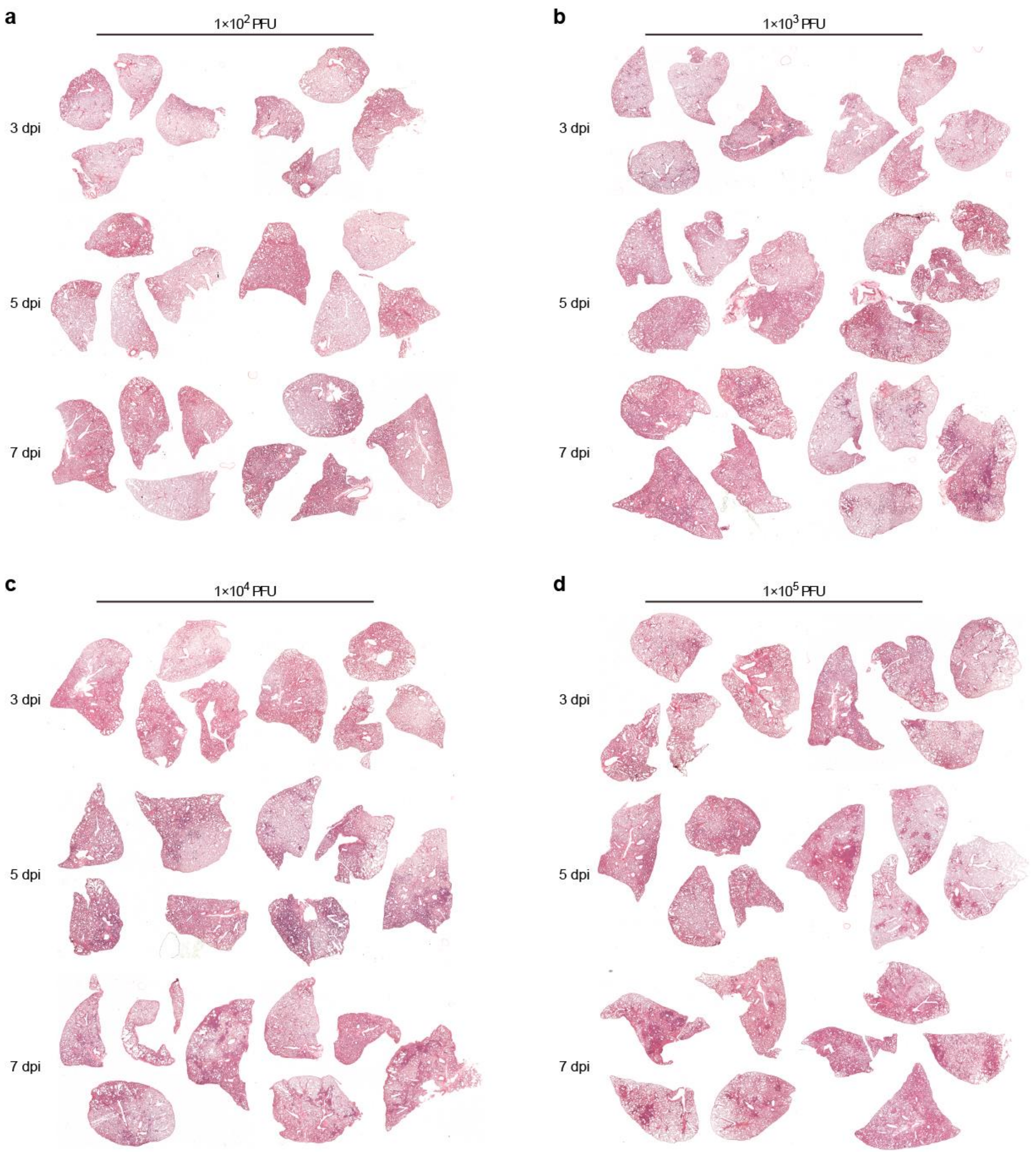

Supplementary Fig. 19. H&E staining for all lung sections collected from individual female hamsters challenged with a dose gradient of **(a)** 1×10^2, **(b)** 1×10^3, **(c)** 1×10^4, and **(d)** 1×10^5 PFU of SARS-CoV-2 at 3 dpi, 5 dpi and 7 dpi respectively.

Supplementary Table. 1.

Details for pathological scoring of male and female hamsters intranasally inoculated with $1×10^4$ PFU of SARS-CoV-2.

Group	Date	Pathological lesions			Comprehensive pathological score
		Alveolar septum hyperplasia and consolidation	Pulmonary edema, hemorrhage and mucus suppository	Recruitment and infiltration of inflammatory cells	
Female 10e4 PFU	3dpi	2+1+1	0+0+0	0+1+1	2+2+2
		2+2+0	0+0+0	1+1+0	3+3+0
		2+1+1	0+0+0	1+1+0	3+2+1
		1+1+1	2+2+1	1+1+1	4+4+3
	5dpi	1+2+1	0+1+0	1+2+1	2+5+2
		1+1+2	0+1+1	1+1+1	2+3+4
		2+1+1	0+1+1	1+1+1	3+3+3
		1+1+2	0+1+1	1+1+1	2+3+4
	7dpi	2+1+1	1+1+1	1+1+1	4+3+3
		2+1+2	1+1+1	1+1+2	4+3+5
		3+2+2	1+1+1	2+2+2	6+5+5
		1+2+1	2+2+1	3+3+1	6+7+3
Male 10e4 PFU	3dpi	1+1+0+0	2+2+1+1	1+1+1+1	4+4+2+2
		3+2+2	2+2+2	3+2+2	8+6+6
		2+1+0	3+2+0	1+2+1	6+5+1
	5dpi	3+3+3+3	2+2+1+1	3+3+3+2	8+8+7+6
		3+3+3+3	1+2+2+1	3+3+2+2	7+8+7+6
		1+1+3+3+3	2+1+1+2+2	2+1+1+3+3	5+3+5+8+8

	**7dpi**	3+3+3+4+4	3+2+1+2+2	4+2+2+4+4	10+7+6+10+10
		2+4+4+4	2+3+3+3	3+4+3+3	7+11+10+10
		3+2+3+3	2+1+1+2	3+2+2+3	8+5+6+8

Supplementary Table. 2.

Details for pathological scoring of male hamsters intranasally inoculated with 1×10^2 to 1×10^5 PFU of SARS-CoV-2.

Group	Date	Pathological lesions			Comprehensive pathological score
		Alveolar septum hyperplasia and consolidation	Pulmonary edema, hemorrhage and mucus suppository	Recruitment and infiltration of inflammatory cells	
10e2 PFU	3dpi	1+2+2	0+1+1	1+2+1	2+3+4
		2+1+2	1+0+1	1+0+2	4+1+5
	5dpi	2+2+4	1+1+2	2+2+4	5+5+10
		1+4+1	1+3+1	1+4+1	3+11+3
	7dpi	3+3+2	2+2+2	3+3+2	8+8+6
		2+3+4	2+2+2	2+3+4	6+8+10
10e3 PFU	3dpi	2+2+2	1+1+2	1+1+1	4+4+3
		3+2	2+2	2+2	7+6
	5dpi	4+3+2	2+2+1	4+3+1	10+8+4
		2+2+4	1+2+2	1+2+4	4+6+10
	7dpi	2+2+3	3+3+3	2+3+4	7+8+10
		3+3+4	3+3+1	2+3+4	8+9+9
10e4 PFU	3dpi	2+2+3	1+2+3	1+1+3	4+5+9
		1+2+2	2+2+1	1+2+3	4+6+6
	5dpi	3+3+2	2+1+1	3+3+2	8+7+5
		2+2+2	2+2+1	2+2+1	6+6+4
	7dpi	4+1+3	2+1+2	4+2+3	10+4+8

		4+4+4	3+2+2	3+4+3	10+10+9
**10e5 PFU**	**3dpi**	2+2+3	1+2+3	1+1+3	4+5+9
		1+2+2	2+2+1	1+2+3	4+6+6
	**5dpi**	3+3+2	2+1+1	3+3+2	8+7+5
		2+2+2	2+2+1	2+2+1	6+6+4
	**7dpi**	4+1+3	2+1+2	4+2+3	10+4+8
		4+4+4	3+2+2	3+4+3	10+10+9

Supplementary Table. 3.

Details for pathological scoring of female hamsters intranasally inoculated with 1×10^2 to 1×10^5 PFU of SARS-CoV-2.

Group	Date	Pathological lesions			Comprehensive pathological score
		Alveolar septum hyperplasia and consolidation	Pulmonary edema, hemorrhage and mucus suppository	Recruitment and infiltration of inflammatory cells	
10e2 PFU	3dpi	0+0+0+0	1+1+0+1	0+0+1+1	1+1+1+2
		0+1+1+1	1+1+1+1	0+0+0+0	1+2+2+2
	5dpi	2+1+1+1	1+1+0+0	1+1+0+0	4+3+1+1
		2+0+0+2	1+0+0+1	1+0+0+1	4+0+0+4
	7dpi	2+2+2+0	1+1+1+0	1+1+0+0	4+4+3+0
		2+2+1+1	1+1+1+1	0+0+0+0	3+3+2+2
10e3 PFU	3dpi	0+0+0+1	1+1+1+2	1+0+1+2	1+2+3+5
		0+0+1+0	0+0+1+1	0+0+1+1	0+0+3+2
	5dpi	1+1+0+1	1+1+0+1	1+1+1+0	3+3+1+2
		0+1+1+1	1+2+2+2	0+2+1+2	1+5+4+5
	7dpi	0+0+0+2	1+1+1+2	1+1+1+3	2+2+2+7
		1+1+1+2	1+1+2+2	1+1+2+2	3+3+5+6
10e4 PFU	3dpi	2+2+2+0	1+1+1+1	0+0+0+0	3+3+3+1
		1+1+1+0	1+1+1+1	0+0+0+0	2+2+2+1
	5dpi	1+1+1+1	2+2+2+1	1+1+1+1	4+4+4+3
		0+1+1+1	1+2+2+2	1+1+2+2	2+4+5+5
	7dpi	1+1+2+1	2+2+3+1	2+1+3+2	5+3+8+5

		1+2+2+1	1+2+2+2	0+2+3+2	2+6+7+5
**10e5 PFU**	**3dpi**	0+1+0+1	1+2+2+1	1+1+1+1	2+4+4+3
		1+1+0+0	1+2+2+1	1+2+1+1	3+5+3+2
	**5dpi**	1+1+1+1	1+2+2+2	1+1+2+2	3+4+5+5
		2+1+1+1	3+1+1+1	4+2+1+1	9+4+3+3
	**7dpi**	3+1+1+1	3+2+2+1	4+2+2+1	10+5+5+3
		3+2+2+1	3+2+2+1	2+2+2+1	8+6+6+3

Description for the contents of the videos:

Video 1: Male hamster intranasally inoculated with 1×10^4 PFU of SARS-CoV-2 showed severe weakness, piloerection, hunched-back and abdominal respiration at 7 dpi.

Video 2: Female hamster intranasally inoculated with 1×10^4 PFU of SARS-CoV-2. The hamsters were sleeping normally. Symptoms such as weakness, piloerection, hunched-back and abdominal respiration were not observed at 7 dpi.

Video 3: Mock hamsters without SARS-CoV-2 infection were health and active. Symptoms such as weakness, piloerection, hunched-back and abdominal respiration were not observed.
